# Multiple clinical risks for cannabis users during the COVID-19 pandemic

**DOI:** 10.1186/s13722-021-00214-0

**Published:** 2021-01-20

**Authors:** Ellen Mello Borgonhi, Vanessa Loss Volpatto, Felipe Ornell, Francisco Diego Rabelo-da-Ponte, Felix Henrique Paim Kessler

**Affiliations:** 1 Center for Drug and Alcohol Research, Hospital de Clínicas de Porto Alegre, Universidade Federal Do Rio Grande Do Sul, Street Professor Álvaro Alvim, 400. Zip-code: 90420-020, Porto Alegre, RS Brazil; 2 Graduate Program in Psychiatry and Behavioral Sciences, Universidade Federal Do Rio Grande Do Sul, Porto Alegre, RS Brazil; 3 Laboratory of Molecular Psychiatry, Hospital de Clínicas de Porto Alegre, Universidade Federal Do Rio Grande Do Sul, Porto Alegre, Brazil; 4 Department of Psychiatry and Legal Medicine, Universidade Federal Do Rio Grande Do Sul, Porto Alegre, Brazil

**Keywords:** Covid19, Cannabis, Clinical risks

## Abstract

The pandemic caused by Sars-CoV-2 (COVID-19) has been a great concern for public and mental health systems worldwide. The identification of risk groups is essential for the establishment of preventive and therapeutic strategies, as for substance users. During COVID-19 pandemic, there was an increase in the use of psychoactive substances during the lockdown, including cannabis. This commentary reviews relevant findings and discusses scientific evidence on the risks of worse clinical and psychiatric complications due to coronavirus disease COVID-19 in subjects who use cannabis. Although they are not included as a risk group in the health recommendations for that disease, they may have a more vulnerable respiratory system to viral diseases. There are certain similarities between the harmful cardiovascular and respiratory effects of cannabis use and those of smoking. Due to the different modes of smoking, cannabis chemicals are retained in the body for longe and may also contain other toxic substances such as tar, a substance found in tobacco and which has been associated with the development of lung cancer, bronchitis and pulmonary emphysema. Therefore, we discuss if individuals who use cannabis regularly might be more vulnerable to COVID-19 infection. This population deserves more clinical attention worldwide and this manuscript can help clinicians become more aware of cannabis risks during pandemics and develop specific intervention strategies.

## Introduction

The pandemic caused by Sars-CoV-2 (COVID19) has caused the collapse of health systems worldwide. Alongside the clinical risk, the appearance or intensification of psychiatric symptoms has also been generating a mental health pandemic within another. The identification of risk groups is essential for the establishment of preventive and therapeutic strategies, for people who are infected by the COVID-19 virus, as well as for users os psychoative substances (PS). During the COVID-19 pandemic, it was identified that the consumption of PS, such as tobacco, cannabis, and cocaine increased the risk of contamination of influenza and was associated with a worse clinical prognosis [1]. A study found that patients with a recent use of PS were at significant risk greater risk of developing COVID-19 compared to patients without a recent substance use disorder (SUD) diagnosis, after adjusting for age, sex, race and types of insurance. Although cannabis users have a lower odds ratio than other drugs (opioids, tobacco, alcohol and cocaine) the risk is five times greater than in people who do not use PS [2]. Thus, cannabis users can be at risk of clinical complications if infected with COVID-19 due to deteriorating health status.

A review conducted in 2019 found that with the increasing increase in cannabis use in the population there has been an increasing number of studies associating cannabis use with serious and life-threatening cardiovascular complications, including acute coronary syndromes, potentially lethal cardiac arrhythmias and ischemic strokes. There are certain similarities between the harmful cardiovascular and respiratory effects of cannabis use and those of smoking. Despite the difference in active ingredients (tetrahydrocannabinol vs. nicotine), along with, due to the different modes of smoking, cannabis chemicals are retained in the body for longer [3]. Furthermore, cannabis may also contain other toxic substances such as tar, a substance found in tobacco and which has been associated with the development of lung cancer, bronchitis and pulmonary emphysema [4]. Although, it is necessary to consider that cannabis is widely used by young populations.

A recent review shows a significant association between cannabis use and symptoms of chronic bronchitis after adjustment for tobacco. Some studies have found a modest reduction in specific airway conductance in relation to marijuana, probably reflecting endoscopic evidence of bronchial mucosa edema among habitual cannabis smokers [5]. The immunosuppressive effects of delta-9 tetrahydrocannabinol (THC) increase the possibility of an increased risk of pneumonia, but further studies are needed to assess this potential risk. Several case series have demonstrated pneumothorax / pneumomediastinum and bullous lung disease in cannabis smokers, but these associations require epidemiological studies for firmer evidence of possible causation. Another recent study reports that the use of vaping causes lung injuries similar to COVID-19 symptoms [6].

Moreover, most of the people who use cannabis also report tobacco use concurrently. This fact may increase the risk of exposure to toxic substances, as certain co-users (for example, blunt users) tend to have higher levels of carbon monoxide in exhaled air and cannabis smoke may have higher levels of some carcinogens than tobacco smoke [7]. Previous studies have indicated that long-term cannabis use is associated with high mortality rates, heart disease [8], metabolic syndromes and changes in the immune system [9]. In this sense, when compared to the general population, we can consider that this population may be more vulnerable to infection morbidity and mortality.

In addition to worsening the clinical outcome (especially involving respiratory risk) [10], cannabis use can generate or increase psychiatric disorders (mainly psychotic symptoms) [11], intense emotional and behavioral reactions were reported during the pandemic, such as fear, boredom, loneliness, anxiety, insomnia, anger and aggression [12]. These symptoms might be especially intense in people who use cannabis, as it is a population that has high rates of psychiatric comorbidities, is particularly sensitive to dysphoric emotional states and has a low tolerance for frustration and stress [13]. Futhermore, it has been described that people who use cannabis may have high rates of psychiatric disorder comorbidities such as simultaneous substance use (mainly tobacco and alcohol), anxiety disorders, mood disorders and personality disorders, potentiating higher risk symptoms in the current scenario [14].

A study conducted in the USA found that 36% of adults had symptoms of anxiety disorders compared to 8% of adults in January–June 2020 (N = 17,067). Anxiety can lead to increased substance use, beginning to use SPA, relapses or even increasing the amount of SPA used. [15]. Another study, carried out with 1054 Canadian adolescents, for a previous period and after the beginning of the practices of social distancing, showed that there was an increase in the use of alcohol and cannabis by this population and although 43% of the sample reported using cannabis and alcohol alone, 23% of the sample reported using it with friends during the period of social isolation, thus placing adolescents at risk of contracting COVID-19. Besides, solitary substance use in adolescence during the pandemic, which is associated with mental health problems and coping, can also be a notable concern, worthy of further investigation [16].

Nevertheless, recent studies also report that the use of cannabinoids offers good results in the treatment of COVID-19, although the endocannabinoid system (ECS) is involved in the regulation of several physiological processes, including sleep and the immune response, its role during infections has not been fully studied. It is well known that the use of this substance increases the susceptibility to infections due to the impact on the modulation of the immune system. Regarding the medicinal or recreational use of cannabis, its influence on the course of an infection, whether caused by bacteria, viruses, parasites, and fungi, has been reported. In this sense, there is evidence to suggest the involvement of ECS in the control and elimination of infectious agents but few studies are available to date. The question therefore arises as to whether ECS increases the severity of viral or bacterial infections and whether consumption of cannabis or synthetic cannabis derivatives / products can influence this risk.

The ECS has a modulating effect on the immune system, but subjects who take cannabinoids or cannabis are not considered immunosuppressed [17]. There are currently no studies available on the incidence and course COVID-19 infection in individuals taking cannabinoids. Pre-existing non-medical consumption of cannabinoids should not be increased, but decreased, during the COVID-19 pandemic, considering potential respiratory complications. One should avoid sharing cigarettes, pipes, or any equipment used for the use of cannabis, as well as any psychoactive substance, as it might be a potential form of transmission of the virus, since the greatest form of dissemination is through droplets of saliva. Likewise, cannabidiol (CBD) cannot be recommended as a possible medication, as the impact on immunity in the case of COVID-19 infection is unclear [18].

In this context, it is essential that this population is recognized according to their clinical and psychiatric vulnerability. It is vital to think about prevention and protection strategies, as well as the recovery of clinical and mental symptoms. In order to prevent the period of social distance from intensifying consumption and all other risks related to it, several measures must be taken. Among them, one must consider: the information must be used as a form of prevention, damage reduction policy, pharmacological approach, brief intervention, possibly online, during the most critical peak of the pandemic. Therefore, it is relevant to refer these patients to highly complex health treatment centers to evaluate and treat them according to the specificity of this population. The clinical and psychiatric aspects of addiction and its relationship with COVID-19 should be noted. There are few studies conducted to date on the increased risk of COVID-19-related morbidity and mortality in this population, although the gray literature has shown data showing the increase in cannabis use in the United States during this period. Epidemiological studies should be carried out to assess a potential increase in cannabis use by the population and investments should be intensified, as well as directed to psychoeducation, to preventive and therapeutic approaches to this substance and its consequences, enhanced by the inherent pandemic risks.

## Conclusion

Our manuscript contains clinically relevant notes on the risks of worse clinical complications due to COVID-19 in marijuana users. Although not included as a risk group in global health recommendations, marijuana users may be more vulnerable to contagion and worsening of their clinical condition due to covid-19 infection are at high risk, this can be explained both by the action of the psychoactive substance on central nervous system and not immune system, as well as due to the method of use that this substance is usually used. In addition, the behavior of marijuana users can make them even more vulnerable to SARS-CoV-2 infection. At this stage that Pandemia is in, with increasing rates of morbidity due to contagion by COVID-19, this population deserves more clinical attention worldwide. Therefore, our letter manuscript can help doctors become more aware of this group and can develop a specific approach.

## Data Availability

Does not apply to this study.

## Abbreviations

PS: Psychoative substances
SUD: Substance use disorder
THC: Delta-9 tetrahydrocannabinol
ECS: Endocannabinoid system
CBD: Cannabidiol
